# Neurodevelopmental delay: Case definition & guidelines for data collection, analysis, and presentation of immunization safety data

**DOI:** 10.1016/j.vaccine.2019.05.027

**Published:** 2019-12-10

**Authors:** Adrienne N. Villagomez, Flor M. Muñoz, Robin L. Peterson, Alison M. Colbert, Melissa Gladstone, Beatriz MacDonald, Rebecca Wilson, Lee Fairlie, Gwendolyn J. Gerner, Jackie Patterson, Nansi S. Boghossian, Vera Joanna Burton, Margarita Cortés, Lakshmi D. Katikaneni, Jennifer C.G. Larson, Abigail S. Angulo, Jyoti Joshi, Mirjana Nesin, Michael A. Padula, Sonali Kochhar, Amy K. Connery, for The Brighton Collaboration Neurodevelopmental Delay Working Group

**Affiliations:** a University of Colorado School of Medicine, Aurora, CO, USA; b Children’s Hospital of Colorado, Aurora, CO, USA; c Department of Pediatrics, Baylor College of Medicine, Houston, TX, USA; d Department of Women and Children’s Health, Institute of Translational Medicine, University of Liverpool, Liverpool, UK; e University of New Mexico School of Medicine, Albuquerque, NM, USA; f Wits Reproductive Health and HIV Institute, University of the Witwatersrand, Johannesburg, South Africa; g Kennedy Krieger Institute, Baltimore, MD, USA; h Johns Hopkins University School of Medicine, Baltimore, MD, USA; i University of North Carolina School of Medicine, Chapel Hill, NC, USA; j Department of Epidemiology and Biostatistics, Arnold School of Public Health, University of South Carolina, Columbia, SC, USA; k Clinical Sciences Latam Sanofi Pasteur, Bogota, Colombia; l Medical University of South Carolina, SC, USA; m Department of Physical Medicine and Rehabilitation, University of Michigan, Ann Arbor, MI, USA; n Center for Disease Dynamics Economics & Policy, Amity Institute of Public Health, Amity University, India; o Division of Microbiology and Infectious Diseases, National Institutes of Allergy and Infectious Diseases, National Institutes of Health, Bethesda, MD, USA; p Children’s Hospital of Philadelphia, Department of Pediatrics, Philadelphia, PA, USA; q Global Healthcare Consulting, India; r University of Washington, Seattle, USA; s Erasmus University Medical Center, Rotterdam, the Netherlands

**Keywords:** Neurodevelopmental delay, Adverse event, Maternal immunization, Guidelines, Case definition

## 1 Preamble

### 1.1 Need for developing case definitions and guidelines for data collection, analysis, and presentation for neurodevelopmental delay as an adverse event following immunization

Neurodevelopmental delay (NDD) is a term used to describe delays in skill development of infants and young children. Terminology and definitions of NDD vary broadly in the literature, though all are used to signify a delay in one or more developmental domains compared to typical development. This paper focuses on defining NDD to assist in the identification of developmental delays as potential adverse events following maternal immunization.

#### 1.1.1 Neurodevelopment

Early child neurodevelopment refers to the organization and function of the central nervous system (CNS). The development of the CNS starts early in embryonic life and continues for years after birth. Processes, such as dendritic pruning, myelination, and the growth of an extensive and complex system of connections accelerate during early childhood and persist into adulthood [1]. The early rapid and complex development of the brain underlies the functional or observable performance and abilities in the child.

The pattern and timing of neurodevelopmental skill attainment is similar for most children [1]. For example, during the first three years of life, early development is marked by enormous gains in gross motor abilities (e.g., rolling, standing, walking), fine-motor coordination (e.g., self-feeding, pincer grasp, drawing lines and circles), language abilities (e.g. orienting to familiar voices, babbling, following a simple command, first words, expanding vocabulary), and markedly improved ability to solve increasingly complex problems. The development of each skill influences the development of others. For example, gross motor skill development allows children greater opportunities for exploration and social interaction which promotes language growth [2], [3], [4]. Likewise, increased language capacity promotes the development of cognitive control [5], [6].

Both biological and environmental factors can influence neurodevelopmental trajectories in positive and negative ways throughout the lifespan, beginning as early as the embryonic period, with observable differences in development and functioning notable as early as the antenatal period [7]. For example, genetic disorders, chromosomal abnormalities, infections, perinatal brain injuries, and alterations in neuronal migration may impact brain development and result in NDD. Poverty, insufficient cognitive stimulation, and malnutrition are significant environmental risk factors for atypical neurodevelopment [8]. Ultimately, biological and environmental factors interact. For instance, environmental risk factors can exacerbate existing biological vulnerabilities, or environmental factors can improve neurodevelopmental outcomes despite biological vulnerabilities. Environmental factors such as good nutrition, environmental stimulation, and maternal factors, including maternal education level and responsiveness, are particularly protective and support a positive developmental trajectory [9].

There are specific time periods in which the brain is more sensitive or vulnerable to biological and environmental influences which can affect the long-term trajectory of the developing brain [10], [11]. This is particularly the case during gestation and early life [12], [13]. For example, alterations in neuronal migration in utero have been associated with movement problems, developmental dyslexia, and other developmental delays [14], [15]. Zika virus is a recent example of devastating developmental sequelae to brain development from in utero infection resulting in microcephaly and potentially more subtle effects if the virus is contracted later in life [16]. Similarly, many studies have found an association between responsive caregiving in sensitive periods of early childhood with improved self-regulatory skills later in life [17], [18].

A NDD is therefore the result of atypical central nervous system development that can occur at any point in utero through the early developmental period up to approximately 5 years of age. The effects of atypical neurodevelopment may impact one area of functioning (e.g., language) or multiple areas (e.g., motor, language, and cognitive). The appropriate and accurate identification of NDDs in infants and young children has important implications for the individual and for public health, including the ability to efficiently allocate available resources, implement early preventive and therapeutic interventions, and monitor health interventions and programs[19].

#### 1.1.2 Cultural and universal determinants of neurodevelopment

Developmental psychology has long debated how a child’s neurodevelopmental trajectory is influenced by biological determinants (i.e., nature) and how much is environmentally influenced (i.e., nurture). More specifically, can developmental skill expectations be universally applied or are they primarily determined by culture and experience? Research has demonstrated that the answer is both nature and nurture, as well as their interplay, with some areas of neurodevelopment more or less influenced by the context of the child’s culture and social practices than other areas [20]. For example, motor skills tend to develop more similarly across cultures [21], whereas the development of social skills may be more variable and dependent on the cultural context, norms, and expectations [20], [22].

Multiple studies have demonstrated the influence of culture on neurodevelopmental skill development [23], [24]. For example, a child’s language skills in the school years are correlated with responsive caregiving and the amount of direct speech input the child receives in the first years of life [25]. In turn, the amount of speech a caregiver directs to his/her child in those early years is influenced by the cultural beliefs around child rearing and the socialization of children. Clearly, cultures vary in the importance placed on certain skills, which subsequently influences both the amount of opportunities available to a child for practice and also the level of adult support provided to foster development of those skills. This, in turn, may influence the trajectory of that skill or the development of that skill at the expense of another [26]. For example, some cultures place a value on skills such as numeracy and writing in school and in the workplace. Therefore, the development of these skills may be prioritized by caregivers more in the early years over the development of others, such as integrating well into the community, which may be considered more important and relevant in another cultural context [27].

Although culture clearly influences developmental skills, studies also show that development usually follows the same progressive patterns globally even though the ages of attainment may vary [22], [28], [29], [30], [31], [32]. Additionally, when sufficient opportunity and stimulation are present and developmental risk factors are minimized, the commonalities across cultural groups in developmental skills increases. A series of studies conducted in Peru, Canada and India have demonstrated that when risk factors are minimized, children between the ages of 1 and 3  years develop similar foundational social-cognitive skills (e.g., imitation, communicative pointing) around the same age across cultural settings [33]. Global commonalities and a universally applied understanding of neurodevelopmental skills may be more applicable in the period of infancy and early childhood than during later years as culture appears to play an increasingly important role [33].

#### 1.1.3 Causes and background rates of NDD

Understanding the incidence of NDD across the world is complicated by several factors. Barriers to assessment and health care access in low- and lower middle-income countries (LMICs), as well as for certain groups in high-income countries (HICs) can complicate the ability to ascertain true incidence rates [34], [35], [36], [37]. Data collection methods can vary substantially and may not include direct measurement, but instead rely on caregiver report of a child’s previous diagnosis of NDD, caregiver report on a developmental screening measure, or other governmental data, such as service utilization [34], [36], [38], [39], [40], [41], [42], [43]. Lastly, many studies reporting on rates of NDD include a large age range or other neurodevelopmental and medical disorders and sensory and motor impairments, which are beyond the scope of this paper and our definition of NDD, making age and domain specific information more difficult to obtain [34].

Due to these issues, it can be difficult to compare rates of NDD even in HICs with presumably greater resources. In the United States between 2014 and 2016, an estimated 4.67% of children ages 3–7  years were identified as having a NDD, excluding children with neurodevelopmental disorders, such as autism and intellectual disability [38]. In the United Kingdom, using a broader definition of developmental delay for a cohort of children born between 2000 and 2002, an estimated 10% of children 9  months of age had mild delays with an additional 2% identified as having more severe delays [44]. Data from 2009 in Australia estimated 7.0% of children ages 0–14 years met criteria for a broader definition of disability encompassing developmental delays, medical problems and sensory and motor impairments [45]. Again, these estimates vary according to definition of NDD, method of data collection, and ages of the children studied.

A paucity of data regarding rates of neurodevelopmental delay in LMICs has resulted in model-based estimates of burden. Current estimates suggest that approximately 43% of children under 5  years living in LMICs fail to reach their cognitive potential because of factors such as poverty, illness, poor nutrition, and lack of stimulating care [46]. Data collected on the Early Childhood Development Index (ECDI) between 2005 and 2015 from almost 100,000 3 and 4-year-old children in 35 LMICs showed 14.6% of children performing poorly in the cognitive domain, 26.2% in the socioemotional domain and 36.8% of children performing poorly on either or both domains. Children living in Sub-Saharan Africa, South Asia, East Asia and the Pacific region were at the greatest risk [47].

It is clear that the risk varies substantially for children depending on where they live in the world and that estimates of neurodevelopmental problems in LMICs are much higher than in HICs because of the increased exposure to these risk factors. While rates of poverty have decreased globally [48], which should lower the risk for NDD, high rates of NDD remain in many parts of the world where few resources are available for screening, treatment and intervention [19]. Advances in medical care have contributed to reduced rates of NDD in regions in which they are available. In parts of the world in which this level of care cannot be accessed, ongoing risk factors remain. For example, improved antenatal, perinatal, and neonatal care and access to early treatment of various infections, such as cerebral malaria or meningitis have contributed to better developmental outcomes [36], [49], [50]. Conversely, NDD rates have risen in some parts of the world where better access to medical care has contributed to increased rates of survival, including for premature birth and for some infections, such as HIV [37], [38], [51], [52]. Some common causes of NDD are described in Table 1.

**Table 1:** Causes of neurodevelopmental delay.

Condition Category	Examples
Perinatal and neonatal events [53]	• Maternal factors [54], [55] (hypothyroxinemia, gestational diabetes) • Premature birth [56] (brain volume changes, white matter injury, intraventricular hemorrhage, IUGR, chronic lung disease) • Low, very low, or extremely low birth weight [57] • Intrauterine growth restriction [58] • Intra-partum related brain injury [59] (hypoxic-ischemic encephalopathy, hemorrhage, perinatal arterial ischemic stroke) • In-utero and Neonatal infections [60] (e.g., Zika [61], Rubella, Varicella zoster, CMV, GBS, E. coli, Listeria, etc) • Increased unbound bilirubin levels [62] • Neonatal hypoglycemia • Exposure to maternal toxins [63] (e.g., fetal alcohol syndrome, narcotics, opioids) • Disorders of neuronal migration [15]
Post-neonatal infections	• HIV infection [64], [65] • Tuberculosis (TB) [66] (Meningitis, tuberculomas, post infection hydrocephalus) • CNS infections [67] (Viral, bacterial : Meningitis, meningoencephalitis, encephalitis, brain abscess) • Post-infectious sequelae (e.g. Sub-acute sclerosing pan-encephalitis, vasculitis) • Malaria [68] (e.g. cerebral or severe systemic malaria complicated by seizures) • Parasitic infection [69] (e.g. neurocysticercosis)
Neurological disorders	• Congenital (Neuronal migration disorders, neural tube defects, hydrocephalus) • Acquired (Stroke, vasculitis)
Genetic abnormalities/syndromes	• Down syndrome • Syndromic and non-syndromic X-linked disorders (e.g. Fragile X syndrome, adrenoleukodystrophy, Rett syndrome) • Autism spectrum disorder • Prader-Willi syndrome • Velocardiofacial syndrome • Neurodegenerative diseases (e.g., sphingolipidoses, neuronal ceroid lipofuscinosis, sialidosis, etc.)
Other Congenital Abnormalities	• Congenital heart disease [70] • Congenital hypothyroidism (untreated) [71]
Inborn errors of metabolism	• Disorders of glycosylation • Disorders of cholesterol metabolism (e.g., Smith-Lemli Opitz) • Disorders of creatinine metabolism • Glycogen storage disorders • Organic acid disorders • Lysosomal storage disorders • Mitochondrial disorders
Other childhood diseases	• Epilepsy [70] • Acquired hypothyroidism [71]
Nutritional abnormalities [46], [72], [73], [74]	• Malnutrition [19], [47] • Micronutrient deficiencies [75]
Toxin exposures [75], [76], [77]	• Lead, mercury, organophosphate poisoning
Other medical	• Repeated early childhood anesthesia/surgery [78] • Cardiac and non-cardiac surgery [79], [80] • Post neurological trauma
Other environmental	• Poverty [19], [46], [47], [75] • Reduced access to clean water [75] • Maternal depression [46], [75] • Insufficient cognitive stimulation [47], [75] • Trauma and exposure to adverse experiences [75], [81]

#### 1.1.4 Medical evaluation of causes of developmental delay

With a broad spectrum of potential causes for developmental delay, a comprehensive medical evaluation can provide insights to commonly associated conditions. Physical exam findings may make certain diagnoses (e.g., Down Syndrome) evident in the newborn period, while other diagnoses (e.g., Fragile X) may be diagnosed at a later age. Genetic testing may include analysis via chromosomal microarray, whole exome sequencing, or karotype. Evaluation for various inborn errors of metabolism may include testing of blood and urine specimens. Neuroimaging (e.g., magnetic resonance imaging) may provide utility for the identification of neurologic abnormalities and/or injury. Additionally, delays in specific domains, such as speech and language, may be closely related to other medical conditions, such as hearing deficits. A complete description of the medical assessment of infants and children with developmental delays is beyond the scope of this review, and guidance is available elsewhere [82].

#### 1.1.5 Limitations of neurodevelopmental assessment in LMIC

Much of the difficulty in gathering data about rates of NDD globally is related to barriers in assessment. While resources to identify and evaluate NDD in children 0–5 years of age are often available in HICs, barriers in health systems and infrastructure, lack of appropriate assessment tools, and other child and family factors challenge this process in LMICs.

Within health systems in LMICs, there is often a shortage of appropriately qualified providers to conduct standardized assessments. Furthermore, most medical professionals, who are most often the primary health care providers of children, are trained to treat acute childhood illnesses, but not to evaluate and recognize NDDs [37]. When available, providers with training in early childhood assessment are often located in larger cities resulting in additional barriers for children living in rural areas [83]. Providers may also feel ethically challenged with diagnosing neurodevelopmental problems when there are limited options for treatment or intervention [83], [84]. Compounding difficulties related to qualified examiners, the health care infrastructure infrequently provides ideal environments (e.g., quiet and private space with limited distractions) to conduct neurodevelopmental assessments.

The lack of availability of neurodevelopment assessment measures in LMICs is also a significant barrier to assessment. Tools may not be available in the local language, and when available, may not have adequately established normative data. Most available measures have been developed in HICs [85], and translated for use in LMICs [34]. Therefore, cultural relevance with regard to test content and materials, as well as methods of administration may not be adequately addressed [34], [86], [87]. This can amplify cultural differences and limit the validity of test findings [88], [89], [90], [91], [92]. Additionally, the psychometric properties of tests developed in HICs, including sensitivity, specificity, reliability, and validity, may be problematic when applied for use in LMICs [34], [93], [94]. Despite these concerns, creating new tests with sound psychometrics, and developing normative standards, is costly and can take several years, especially in LMICs where resources are often limited [34], [95], [96].

Engaging children in direct neurodevelopmental assessment can also prove challenging in LMICs due to their often limited experience completing such tasks and possible anxiety or fear around working with a health professional [93]. Differing cultural beliefs of the provider or family can also impact early recognition or identification of a NDD [85], [97], [98], [99], [100], [101]. Low rates of diagnosis of certain developmental disorders, such as autism spectrum disorders, may be indicative of the community’s “tolerance” for a certain degree of behavior differences, rather than an indication that such behaviors are not observed at comparable rates across cultures [98]. Conversely, in high risk environments where NDDs are highly prevalent, caregivers may not have a reference for normative child development. Other barriers to reliable and valid assessment of neurodevelopment include caregiver literacy/comprehension and concerns around stigmatization, which can limit the use of written questionnaires, checklists, and answers to interview questions [85], [102]. These factors can also influence families’ willingness to bring their children for neurodevelopment assessment and may be a barrier to corroborating findings on standardized assessments with caregiver report [102].

#### 1.1.6 Existing case definitions for NDD

Case definitions of neurodevelopmental delay are provided by the World Health Organization (WHO) and the American Psychiatric Association (APA). Each proposes unique, but related terminology to capture and define NDD.

The *International Statistical Classification of Diseases and Related Health Problems* (ICD) is the medical classification system of the World Health Organization (WHO). Impairment or delay in the maturation of the brain and central nervous system is underscored by the existing ICD-10 definition of NDD, which lists the range of developmental problems under the category of Mental, Behavioral and Neurodevelopmental Disorders and the subcategory of Pervasive and Specific Developmental Disorders. These diagnostic categories include Specific Developmental Disorders of Speech and Language, Specific Developmental Disorder of Motor Functioning, Other Disorder of Psychological Development, and Unspecified Disorder of Psychological Development. Lack of Expected Normal Physiological Development, Lack of Expected Normal Physiological Development, Unspecified, and Delayed Milestone (for walking and talking) are terms used in ICD-10 to capture general or specific developmental delays [103]. The proposed 11th revision defines neurodevelopmental disorders as behavioral and cognitive conditions that emerge during the developmental period with difficulty in the acquisition and execution in specific intellectual, motor, and social domains. The proposed ICD-11 continues to use the term “delayed milestone” to capture delays in social, motor and language domains.

The American Psychiatric Association (APA)’s *Diagnostic and Statistical Manual of Mental Disorders, 5th Edition* (DSM-5) was developed with the goal of harmonization with ICD-11. NDD is addressed in several diagnostic categories. Global Developmental Delay (GDD) is a broader diagnostic description that defines individuals under the age of 5 who fail to achieve developmental milestones in two or more developmental domains at the expected age. A diagnosis of GDD can also be given when global developmental delays are observed, but the child is either too young or unable to undergo systematic testing [104]*.* Delays in specific domains of language and motor are addressed through the diagnostic categories of Communication Disorders (e.g., Language Disorder) and Motor Disorders (e.g., Developmental Coordination Disorder), respectively. Diagnoses of Other Specified Neurodevelopmental Disorder and Other Unspecified Neurodevelopmental Disorder are applied when a NDD is present with an observed impairment in functioning, but the child does not meet criteria or there is not sufficient information for a more specific diagnosis of NDD. [104]*.*

There are some differences among the two major classification systems in the lexicon of the diagnosis of NDD. However, there is substantial overlap regarding the inclusion of different developmental domains and the shared view that these conditions arise during the early developmental period and impact functioning of the child in daily life.

#### 1.1.7 Need for a harmonized definition of NDD

There is no uniformly accepted definition of NDD that can be applied to evaluate outcomes following immunizations in pregnancy. This is a missed opportunity, as data comparability across trials or surveillance systems would facilitate data interpretation and promote the scientific understanding of NDD in the context of the assessment of safety of vaccines for maternal immunization.

In this paper, we provide a case definition for NDD, which focuses on a child’s performance in specific domains. This definition is distinct from neurodevelopmental disorder diagnoses, such as autism, intellectual disability, attention deficit/hyperactivity disorder; medical diagnoses such as cerebral palsy, HIV or specific genetic syndromes; or hearing, vision, or other sensory impairments. Diagnosis of these disorders typically requires a specialized clinical skill set or medical tests. However, presumably, many children with these diagnoses would also meet criteria for a NDD based on the current case definition.

### 1.2 Methods for the development of the case definition and guidelines for data collection, analysis, and presentation for NDD as an adverse event following maternal immunization

Following the process described in the overview paper [105] as well as on the Brighton Collaboration Website http://www.brightoncollaboration.org/internet/en/index/process.html, the Brighton Collaboration *Neurodevelopmental Delay Working Group* was formed in 2018 and included members of clinical, academic, public health, and industry background with varied geographic representation. The composition of the working and reference group as well as results of the web-based survey completed by the reference group with subsequent discussions in the working group can be viewed at: http://www.brightoncollaboration.org/internet/en/index/working_groups.html.

To guide the decision-making for the case definition and guidelines, a literature search was performed using Medline, PubMed, Embase, Cochrane Libraries, Ovid, Springerlink, and Google Scholar including the terms “neurodevelopmental disorder”; “neurodevelopmental disability”; “neurodevelopmental delay”; “neurodevelopmental impairment”; “neurodisability”; “neurodevelopment”; “developmental delay”; “developmental disability”; “global developmental delay”; and “delayed milestones”. The search resulted in the identification of 9394 references. A broader literature search for articles with “neurodevelopmental delay” in the title resulted in 147 articles. Finally, when the term “definition” was added to terms related to NDD, the search resulted in identification of 29 references. All titles and abstracts were reviewed to document the existing definitions of neurodevelopmental delay and methods of evaluation. General medical, neurodevelopmental, pediatric and infectious disease textbooks were also searched. The methods and results of the search to assess the existing literature on maternal immunization and neurodevelopmental delay are described in Section 1.2.1.

Findings from the literature search included a wide range of case reports, survey research, and cross-sectional and longitudinal studies. The terminology for neurodevelopmental delay was inconsistent across studies. There was also a large range in what constituted a “delay” across studies. In many studies NDD was defined by delays in one or more developmental domains (e.g., motor, language). Some studies used the term “developmental delay” and did not provide a case definition. As a result, the workgroup members reviewed 3 case definitions (i.e., ICD-10, ICD-11, DSM-5) in addition to considering common definitions used in research.

#### 1.2.1 NDD following maternal immunization

In order to identify any reported potential association of maternal immunization with infant neurodevelopmental delay (NDD), separate literature searches were performed using Medline, PubMed, the Cochrane libraries, and Embase.com. The results were limited to those in the English language and published in the last 10  years in Embase, while no time or language limits were selected for the other searches. All searches included the terms ‘neurodevelopmental delay,’ ‘developmental delay,’ ‘maternal immunization,’ ‘maternal vaccination,’ ‘pregnancy vaccination,’ ‘antenatal, ‘vaccine’ in conjunction with a specific vaccine, including tetanus (TT, Td, Tdap), pertussis (Tdap), seasonal or pandemic influenza, hepatitis (any), meningococcal, measles, mumps, rubella, MMR, varicella, yellow fever, group B streptococcus (GBS), and respiratory syncytial virus (RSV). These terms were either present as subject headings or in the title or abstracts.

The Medline, PubMed, and Cochrane searches jointly yielded a total of 132 publications. All titles and abstracts were screened for possible reports of NDD following maternal immunization. Most publications referred to the effect of infection in pregnancy on the infant’s development (e.g., maternal rubella, cytomegalovirus (CMV), hepatitis B, or HIV infections), infections in the perinatal period and neurodevelopment (e.g., Group B streptococcus (GBS) infections), or were evaluating the effect of mercury or thimerosal exposures during pregnancy or during childhood vaccination.

Among the results, 5 articles with potentially relevant material were reviewed in detail and summarized in a report including information on the study type, the vaccine, the diagnostic criteria or case definition used, and the clinical description of the cases. Of these, one observational study of tetanus vaccine exposure (Td) did not find an association between maternal immunization and neurodevelopmental delay in their children [106], [107]. One study, focusing on understanding thimerosal exposure from Td vaccine during pregnancy, defined neurodevelopmental delay by using parameters set by the Gesell Developmental Schedules (GDS), which included reflexes, postural reactions, and measures of motor, visual, and auditory development and reactions in response to stimuli, and evaluated exclusively breastfed infants of mothers who received 1–3 doses of Td vaccine. The study concluded that maternal thimerosal exposure in Td vaccines per se was not associated with neurodevelopmental delays measured by GDS in infants at 6  months of age. In one recent study, neither influenza nor Tdap vaccination during pregnancy was associated with increased risk for ASD in infants of vaccinated mothers [107]. In addition, five Cochrane meta-analyses of various vaccines administered during pregnancy, including *Haemophilus influenza* type b, influenza, pneumococcal vaccine, Hepatitis vaccine and tetanus vaccine, did not identify neurodevelopmental issues in infants of vaccinated mothers, although testing tools were not described [108], [109], [110], [111], [112].

The Embase platform search resulted in 96 references overall. All titles and abstracts were reviewed for possible reports of NDD following maternal immunization. Publications in which the NDD discussed in the article was a result of an infection or another known condition and vaccines were only mentioned tangentially, or developmental delay was not actually discussed in the study, were eliminated. A total of 34 articles were identified and reviewed in more detail. Nine book chapters that focused on neurodevelopment or neurodevelopmental disorders were also reviewed. Similar to the prior searches, most publications were evaluating the safety of vaccines in children, not pregnant women, including the effect of vaccine components, such as thimerosal and aluminum in neurodevelopment.

Only 3 articles provided information on vaccines administered during pregnancy. Among these, the same article of Td vaccination in pregnancy described above [106] was identified. The second article was a systematic review of maternal immunization which searched available safety evidence in PubMed and Scopus databases, as well as post-marketing surveillance data, including the Vaccine Adverse Event Reporting System (VAERS) database. This review identified 6 studies on hepatitis B vaccine, 6 on pneumococcal polysaccharide vaccine (PPSV23), and 3 on meningococcal polysaccharide vaccine (MPSV), plus 3 additional studies that compared PPSV with MPSV in pregnant women [113]. Additionally, the study included 91 reports on vaccinations in pregnant women identified from post-marketing surveillance data (88 on hepatitis B, 2 on PPSV, and 1 on MPSV). Overall, NDD in infants of vaccinated mothers was not reported as an event in this systematic review. The third article was a systematic review of the safety of influenza immunization during pregnancy for the fetus and neonate, which summarized 40  years of research on influenza vaccination in pregnant women, and did not identify infant developmental delay as a concern [114].

Recent clinical studies of influenza, Tdap, RSV and GBS vaccines administered during pregnancy have included neurodevelopmental evaluation of infants for a variable period of time, usually 6  months to the second year of life, using various tools such as the *Bayley Scales of Infant and Toddler Development, Third Edition* [115]. No specific associations between maternal vaccination and neurodevelopmental deficits have been identified in these studies [116], [117], [118], [119], [120], [121], [122], [123], [124].

In summary, no evidence of an association between vaccination during pregnancy and NDD in infants and young children was found.

### 1.3 Rationale for selected decisions about the case definition of NDD as an adverse event following maternal immunization

The focus of the working group was to agree on a harmonized definition of NDD and how to identify it with different levels of certainty, which will be useful for the identification of NDD in the context of vaccination of women during pregnancy.

The working group agreed that the term NDD describes a group of developmental problems that begin during the early developmental period and result from impairment in the central nervous system. NDD manifests as developmental functioning that is at least two standard deviations below the norm in one or more of the following domains: gross motor, fine motor, expressive language, receptive language, and/or cognitive/problem-solving.

Levels of diagnostic certainty are suggested by the working group based on two major considerations: 1) the background, training and ability of the evaluator to ascertain the presence of NDD and 2) the methods/tools utilized for assessment (see 1.3.6, 1.3.7). The diagnostic levels of certainty should be applied separately to each domain evaluated. The diagnostic levels must not be misunderstood as reflecting different grades of clinical severity. They instead reflect diagnostic certainty (see Section 1.3.8).

#### 1.3.1 Related term(s) of NDD

Related terms that are commonly used to refer to NDD include global developmental delay, developmental delay, delayed milestones, developmental disability, intellectual disability, neurodevelopmental disability, and neurodevelopmental disorder. Specific delays or disorders in the motor, language, and cognitive are also used (e.g., expressive language delay, gross motor delay).

#### 1.3.2 Domains of neurodevelopmental delay and differentiation from other disorders

A comprehensive neurodevelopmental assessment includes all of the domains, or developmental skill areas necessary to assess the functioning of the child at each phase of development, including early and late infancy, toddlerhood, and early childhood (preschool years). Each skill area has a trajectory of its own and is also influenced by skill development in other areas. The sequence of development in a specific domain is near universal in most cases (e.g., babbling followed by first words), but the rate of skill development in each area may differ. A child may advance within typical timeframes for one domain while lagging behind in another domain at a given age (i.e., dissociation of skills). Therefore, it is imperative to assess for neurodevelopmental delay comprehensively and document if a child is meeting developmental milestones for each specific domain across time points.

For the current case definition, the neurodevelopmental domains of gross motor, fine motor, expressive language, receptive language, and cognitive/problem solving are considered in children 0–5 years of age (see Table 2). The recommended domains correspond not only to the developmental areas outlined in the ICD-10 and ICD-11 definitions of Delayed Milestones [103], but also to the selected domains evaluated on standardized measures of development [125], [126], [127], [128].

**Table 2:** Neurodevelopmental domains included in the NDD case definition.

Domain	Definition	Commonly Used Terms
Motor	Gross-motor skills: large, coordinated body movements (e.g., crawling, walking); Fine-motor skills: small, precise hand movements (e.g., picking something up with hands, using an eating utensil)	Movement, physical development, fine-motor skills, gross-motor skills
Language	Expressive language: ability to express interests, thoughts, needs; Receptive language: ability to understand what others say	Communication, speech, expressive language, receptive language
Cognitive/Problem Solving	Capacity to learn, reason and think in order to solve a problem, explore and play	Thinking, learning, reasoning, visual-reception, non-verbal reasoning

There are additional domains that should be considered in the context of a comprehensive neurodevelopmental assessment, but for which an isolated problem does not meet criteria for the current case definition. These include social, emotional, and behavioral adjustment, as well as daily living skills (also referred to as adaptive behavior). The rationale behind these decisions is explained briefly next.

Age appropriate social, emotional, and behavioral adjustment is dependent on healthy brain development, and a NDD can manifest as difficulties in one or more of these areas. However, for the purpose of the present case definition, we do not recognize an isolated problem in social, emotional, or behavioral functioning for multiple reasons. In this age range, social, emotional, and behavioral functioning are measured primarily with caregiver-report questionnaires. Results can therefore be heavily influenced by contextual factors, including varying cultural expectations, as well as caregiver literacy and comprehension. Practically speaking, existing measures vary widely in the specific skills they assess making global comparisons difficult, and access to well-validated and locally-adapted measures is a barrier in many settings around the world. In the future, as researchers further define the specific social, emotional, and behavioral subskills most likely to result from atypical neurodevelopment in infants and young children and as more reliable and valid measures of those skills become available globally, it could become more appropriate to recognize a single-domain problem in one of these areas as meeting criteria for NDD. For now, clinicians and researchers should at least screen for concerns in these areas whenever possible for two reasons. First, results can provide important information about the validity of other assessment measures. For example, a child who is highly anxious or hyperactive may not participate optimally in the assessment of cognition or language. Second, concerns about social, emotional, or behavioral functioning will often drive clinical management, including intervention recommendations.

Daily living skills (adaptive behavior) are also primarily measured by caregiver questionnaire or interview, and so reliable assessment of this domain is also dependent on the literacy or comprehension of the caregiver. Furthermore, delays in this domain are not typically seen in isolation. Rather delays in daily living skills are usually the result of a delay in another of the outlined neurodevelopmental domains (cognitive, motor, language) and therefore, are not included in the current case definition.

#### 1.3.3 Duration of follow up and age range of neurodevelopmental delay

There are no standard minimum or maximum duration of follow up requirements in studies of maternal immunization [129]. However, in order to determine there has been no adverse developmental outcome and a case of neurodevelopmental delay is not present or does not arise during the period of early childhood, it is recommended that assessment occur in each of the following four time periods: early infancy (0–6  months), late infancy (7–18  months), toddlerhood (19–36  months) and preschool years (37–60  months). While a single time point may be adequate and acceptable for some studies, the strength of any given study will improve with each additional time point added. Additionally, if a single time point is the only available option, choosing which of the four time periods in which to conduct the assessment should be determined by the aims of the study.

When feasible, assessment at multiple time points throughout the period of early childhood is important for several reasons [130], [131], [132]. First, even the most robust, standardized developmental assessments have psychometric limitations at the youngest ages [133]. These include floor effects (i.e., infants have a limited repertoire of skills and therefore the number of test items for younger children is limited), and limitations of test/retest reliability and sensitivity/specificity [133]. Neurodevelopmental re-evaluations over time can improve the ability to detect a NDD and prognosticate long-term outcomes [8]. Second, as previously stated, developmental skill attainment can be variable, such that delays may spontaneously resolve, remit following intervention, or worsen as environmental demands increase and a child fails to acquire the necessary skills to meet these demands [134], [135], [136], [137].

#### 1.3.4 Influence of treatment on fulfillment of case definition

The Working Group decided against using “treatment” or “treatment response” towards fulfillment of the NDD case definition. A treatment response or its failure is not in itself diagnostic, and may depend on variables like clinical status, time to treatment, availability of treatment and other clinical parameters.

#### 1.3.5 Timing post-maternal immunization

A definition designed to be a suitable tool for the eventual assessment of causal relationships requires ascertainment of the outcome (e.g., neurodevelopmental delay) independent from the exposure (e.g., maternal immunizations). Therefore, to avoid selection bias, a restrictive time interval from maternal immunization to onset of neurodevelopmental delay should not be an integral part of such a definition. Instead, where feasible, details of this interval should be assessed and reported as described in the data collection guidelines.

Further, unlike other medical events, there is typically not a sudden onset of a NDD and delays arise outside the controlled setting of a clinical trial or hospital. In some settings, it may be impossible to obtain a clear timeline of the event, particularly in less developed or rural settings. In order to avoid selecting against such cases, this case definition avoids setting arbitrary time frames.

To determine that a case of NDD is present or arises during the period of early childhood, best practice would include continued assessment over the four time periods (early infancy, late infancy, toddlerhood, preschool years), as outlined in Section 1.3.3. However, there are no standard minimum or total duration of follow up requirements in studies of maternal immunization. Therefore, the minimum and total duration of assessment for NDD must be determined for each study, based on the specific needs and characteristics of the study, including the vaccine, the study population, and other relevant factors.

A study with a limited timeframe (i.e., including less than the four suggested time periods) will not limit the researcher’s ability to obtain Level 1 diagnostic certainty. However, it will limit the researcher’s opportunity to identify all potential cases of NDD that may have arisen in the time of early childhood and may have been captured at other time points. Importantly, a case of NDD that is identified at an early time point (e.g., late infancy) and is no longer identified at a later time point (e.g., preschool years) should be considered a documented case of NDD (at the appropriate level of certainty for the early time point) and not as a false positive because delays can spontaneously resolve or respond to treatment/intervention.

#### 1.3.6 Defining the “Gold Standard” – Personnel

This section describes characteristics of gold standard personnel for assessment of NDD. The diagnosis of a NDD is based on observable behavior in the child and requires more subjective judgement than a standard medical test or the developmental assessment of older children, which may provide more objective results. Therefore, diagnostic certainty for a diagnosis of NDD must rely more heavily on the training and background of the evaluator. Gold standard personnel have the training and expertise to select, administer, score, and interpret the measures described below. In general, such professionals will have the highest available level of training in assessment of children aged 0–5 years. In HIC settings, training will typically include a doctoral degree (i.e., physician or psychologist) with specialized training in assessment of infants and young children (e.g., developmental pediatrician, pediatric neuropsychologist, pediatric neurologist, pediatrician or clinical psychologist who has pursued focused training in early childhood assessment). In LMIC settings, there will be cases where the available professional training/credentialing diverges from these examples; professionals in those settings still meet gold standard criteria if they have completed the highest level of training available in their setting and specialize in the assessment of young children. A domain-specific specialist could also meet gold standard criteria if the delay is identified within that single domain. Thus, a speech-language therapist with appropriate training/experience in children aged 0–5 would meet gold standard criteria for diagnosing NDD impacting language, while a physical or occupational therapist with appropriate experience could meet such criteria for diagnosing a NDD impacting motor skills. Gold standard personnel must be fluent in the native language of the child being assessed.

If gold standard personnel are not available, diagnosis of NDD can be made at a lower level of certainty. We discriminate two further categories of personnel, “below gold standard” and “well below gold standard.” A listing of the personnel belonging to each category is provided in Table 3. In general, below gold standard personnel include professionals with similar general background training to gold standard personnel (e.g., physicians, psychologists, domain-specific specialist evaluating that domain), but less comprehensive specialization in early childhood assessment. Nurses and social workers with early childhood assessment training also fit into this category. Well below gold standard personnel include community health workers or the equivalent who lack expertise or have only limited training in child development, psychometrics, and the assessment process.

**Table 3:** Personnel standards.*

Gold standard	• Diagnosis made by an evaluator fluent in the native language with advanced training and expertise in 0–5 assessment and with the highest level of training as defined by the specific setting (e.g., HIC or LMIC). • Diagnosis in a specific domain made by a specialist in that domain (e.g., a speech/language therapist). This specialist should have advanced training and expertise in 0–5 assessment and have the highest level of training as defined by the specific setting.
Below gold standard	• Diagnosis made by an evaluator with advanced training and the highest level of training as defined by the specific setting, but without or less expertise in 0–5 assessment. • Domain specific specialist as defined by the setting (e.g., a speech/language therapist) for each domain assessed without or less expertise in 0–5 assessment. • Nurse or equivalent health care provider, as defined by the highest level of training for the specific setting, with expertise in 0–5 assessment. • Domain specific specialist as defined by the setting with expertise in broad-based 0–5 assessment, or assessment of skills outside of the area of primary expertise (e.g., a speech/language therapist evaluates motor and cognitive functioning).
Well below gold standard	• Diagnosis made by a trained provider or community health worker with at least minimal training in 0–5 neurodevelopmental assessment.

* For the purpose of research studies, personnel can move up one level from the level at which they are placed by their training and background (i.e., from well below gold standard to below gold standard or from below gold standard to gold standard) if they are administering a specific measure on which they have been adequately trained by gold standard personnel.

For the purpose of research studies, personnel can move up one level from the level at which they are placed by their training and background (i.e., from well below gold standard to below gold standard or from below gold standard to gold standard) if they are administering a specific measure on which they have been adequately trained by gold standard personnel. In these cases, it is useful to periodically and formally measure the accuracy and reliability of the trainee (i.e., through interrater reliability, rater accuracy) to ensure they have achieved and have retained mastery of that particular measure.

Additionally, if an interpreter is used, that interpreter should be professional/certified or properly trained as a translator as defined by the setting. When an interpreter is used for the assessment of motor skills, the LOC is determined by the qualifications of the primary evaluator because the primary evaluator is still able to observe and evaluate the child’s performance first hand. However, when an interpreter is used for language and/or cognitive assessment, the LOC moves down one level from the level defined by the qualifications of the primary evaluator. In these cases, the evaluator must rely on the interpreter to translate the child’s responses and so direct assessment by the evaluator of the child is not possible. The assessment measure should also already be in the target language and “live interpretation,” or translation of a testing measure during the assessment, is not recommended. The use of family members for interpretation is also not recommended.

#### 1.3.7 Defining the “Gold Standard” – Assessment

The goal of this section is to describe characteristics of a gold standard assessment tool for NDD in children aged 0–5 years. By “gold standard” we do not mean a perfect assessment, but simply the best that is currently available [138]. In clinical practice, diagnosis should result from a process through which a competent professional integrates test results assessing neurodevelopmental domains with information about the child’s history (typically gleaned from caregiver interviews and any available medical records) and current functioning (gleaned from interviews, observations, and/or caregiver-completed rating scales) [139]. When classifying children for research purposes, the process is similar but will de-emphasize clinical judgment and rely more heavily on specific test scores. Nonetheless, involvement of appropriately trained personnel is important to ensure proper test administration, scoring, and interpretation (see Section 1.3.6).

Robust standardized tests are a pillar of gold standard assessment for NDD. A discussion of specific measures is beyond the scope of this paper; fortunately, several relevant reviews have recently been published [35], [140], [141]. Whenever possible, measures should provide coverage of the five neurodevelopmental domains identified earlier (gross and fine motor, expressive and receptive language, and cognition/problem solving). In almost all cases, these are best assessed with standardized performance-based assessments that are individually administered to the child. Multiple measures are sometimes needed, although some infant and young child assessments encompass all domains [115], [142].

Gold standard assessment measures are characterized by a number of important features. First, they should be psychometrically robust with evidence for good reliability, validity, and sensitivity/specificity for the population and context *in which they are being used* [93]. Reliability encompasses measures of internal consistency, test–retest stability, and inter-rater agreement. General guidelines [143] suggest that for measures of internal consistency (e.g., Cronbach’s alpha) and test–retest stability (e.g., Person’s r), values > 0.7 indicate adequate reliability while values > 0.85 are preferable. For measures of inter-rater agreement (e.g., kappa), values > 0.4 are adequate while values > 0.6 are preferable. Validity is a multi-tiered construct, only some aspects of which can be readily captured statistically. The most straightforward way to establish diagnostic validity of a measure is by comparison to an existing gold standard (i.e., criterion validity), but this approach is not currently feasible in many parts of the world. Convergence with current indicators of daily functioning or prediction of future functioning (e.g., adaptive behavior, academic achievement, or diagnosis of intellectual disability) can also support the validity of neurodevelopmental measures. Sensitivity is the test’s ability to correctly identify patients with NDD, while specificity is the test’s ability to correctly identify those without NDD. Diagnostic assessment should maximize both sensitivity and specificity, with levels of 70–80% or greater generally considered acceptable [144]. An additional psychometric consideration for assessment of NDD in young children is that the measure must include enough easy items to capture variability in the low tail of the distribution (i.e., avoid a floor effect).

A second characteristic of gold standard measures is that they must be linguistically and culturally appropriate for the child and family. Since the most widely used standardized measures have been developed and researched in HICs and originally written in English, assessment of children in LMICs and non-English speaking countries often requires translation and adaptation of existing measures. There is a literature to support using tests developed in HICs to assess children in LMICs when carefully, translated, adapted and applied [145], [146], [147], [148] using specific methodology to ensure they are culturally and linguistically appropriate [149], [150], [151]. Of course, the psychometrics of an adapted test need to be studied and the LOC will be determined by the outcome of those studies (with consideration for the expertise of personnel) and not by the psychometrics of the original version of the test. Therefore, while certainly complex and requiring careful consideration of norming and these other issues, it is possible to attain a high level of diagnostic certainty with an adapted test. Another option available is the creation of a new test. Both adaptation/translation and new test development are complex and challenging and readers are referred elsewhere for a detailed description of the issues involved [140], [152], [153].

Third, gold standard tests must have appropriate norms available, which means that the demographic characteristics of the child being evaluated should be represented in the normative sample. National or regional norms are often preferable [154]. Narrow norms (e.g., drawn from a small local population) are often insufficient for diagnosis of NDD generally as they may obscure the effects of true etiologic risk factors that have negatively impacted a large proportion of children in a locale (e.g., disease, malnutrition, psychosocial adversity, etc.). However, when attempting to isolate the potential effect of maternal vaccination, local norms may be appropriate by controlling for other neurodevelopmental risk factors common to children in that community. Determining which measures are the best available will also rely on practical considerations, such as affordability and access to an appropriate testing environment. In general, measures should be appealing to infants and young children who may have limited experience interacting with strangers and should also be robust to minor fluctuations in the child’s physical/emotional state.

In some circumstances, the only available measure will be a universal or regional developmental milestones checklist. This is not ideal as the psychometric properties of a developmental milestones checklist utilized to diagnose NDD are unknown and are likely substantially weaker than for standardized performance-based testing. Additionally, the demographics of representative children may be too broad to apply to an individual child. However, if a checklist is the only option and is utilized, NDD is diagnosed at a low level of certainty (well below gold standard assessment with consideration for the expertise of the evaluator determining the level), regardless of the severity of NDD identified.

In addition to selecting measures with demonstrated validity at the group level, the examiner must consider factors that can provide a threat to the validity of an individual test administration. In other words, if a child obtains a low score, it should be because of true neurodevelopmental problems rather than uncorrected sensory impairments, inhibited temperament, fatigue, lack of familiarity with test materials/expectations, or other such factors [140]. Personnel with expertise in early childhood assessment should be able to ensure that the child is calm, alert, and engaged before proceeding with standardized testing. In older children, standardized measures are available to objectively assess test engagement [155], but in infants and very young children, clinician judgment is required. Caregiver interview should be conducted to help establish that scores from tests individually administered to the child are valid (i.e., reflective of the child’s typical daily functioning). The evaluator should always include screening for hearing and vision problems (i.e., by asking whether the child has had recent vision/hearing tests, asking caregivers about vision/hearing concerns, and using behavioral observations to gauge whether vision or hearing difficulties appear to interfere with testing). Please refer to the Section 1.3.9 for more detailed information.

The general principles characterizing gold standard assessment of NDD are universal, but the most appropriate specific measures will depend on multiple factors and in many contexts, there will be barriers to an optimal assessment process. If gold standard measures are not available, a diagnosis of NDD can be made at a lower level of certainty (i.e., “below gold standard” and “well below gold standard”). A listing of the assessment standards belonging to each category is provided in Table 4.

**Table 4:** Assessment standards.*

Gold standard	Performance that is 2.0 or more standard deviations below the mean with a test/instrument that is culturally and linguistically appropriate and has strong psychometric properties defined for each domain. For example: • Strong reliability (i.e., internal consistency and test–retest > 0.85; interrater agreement > 0.6) • Strong evidence for validity in the context in which is it being used (e.g., local content experts agree items are culturally and linguistically appropriate; correlates around 0.8 or higher with existing gold standard measure or correlates around 0.8 or higher with current or future measures of adaptive functioning) • Sensitivity and specificity > 0.7–0.8 • Test has norms available with the demographic characteristics of the child being assessed represented in the norming sample
Below gold standard	Performance that is 2.0 or more standard deviations below the mean with a test/instrument that does not meet the gold standard threshold for cultural and linguistic appropriateness and/or has suboptimal psychometric properties defined for each domain. For example: • Adequate reliability (i.e., internal consistency or test–retest > 0.7 but ≤ 0.85, interrater agreement > 0.4 but ≤ 0.6) • Some evidence for validity in the context in which it is being used, but does not meet gold standard criteria • Sensitivity or specificity values < 0.7 • Appropriate norms are not available
Well below gold standard	Performance that is 2.0 or more standard deviations below the mean with a test/instrument that is well below threshold cultural/linguistic appropriateness and psychometrics defined for each domain. For example: • Inadequate reliability, little evidence for validity in the context in which the is being used, or sensitivity and specificity well below < 0.7 • Norms do not include demographic characteristics of the child being assessed

When using a universal or regional developmental milestones checklist, NDD is diagnosed when a minimum delay equal to or greater than half of the child’s age (e.g., a six month delay or more for a child 12 months of age) is found when measured against a universal or regional developmental milestones checklist.

#### 1.3.8 Formulating a case definition that reflects diagnostic certainty: weighing specificity versus sensitivity

It needs to be re-emphasized that the grading of definition levels is entirely focused on diagnostic certainty, not the clinical severity of a NDD. Thus, a clinically very severe NDD may appropriately be classified as Level 2 or Level 3 if Level 1 personnel and procedures were not available. Detailed information about the severity of the event should additionally always be recorded, as specified by the data collection guidelines below.

**Level 1:**
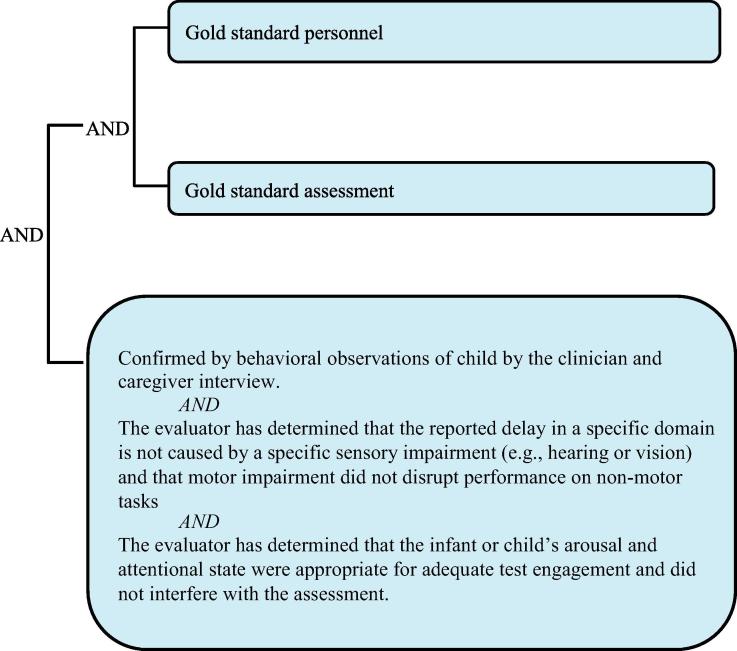
Diagnostic certainty.

**Level 2:**
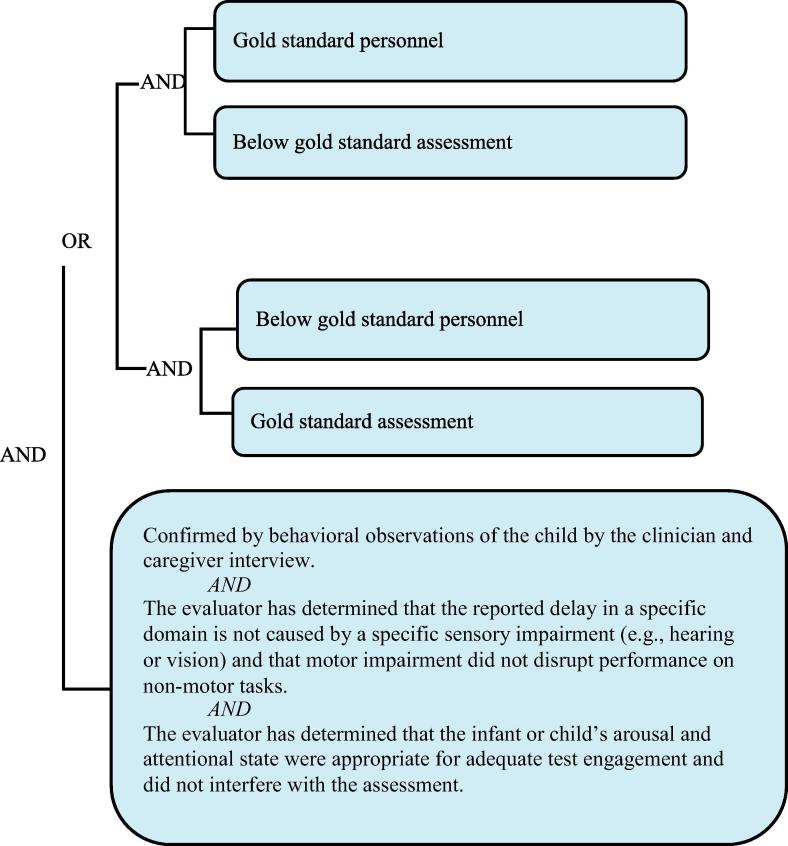
Diagnostic certainty.

**Level 3A:**
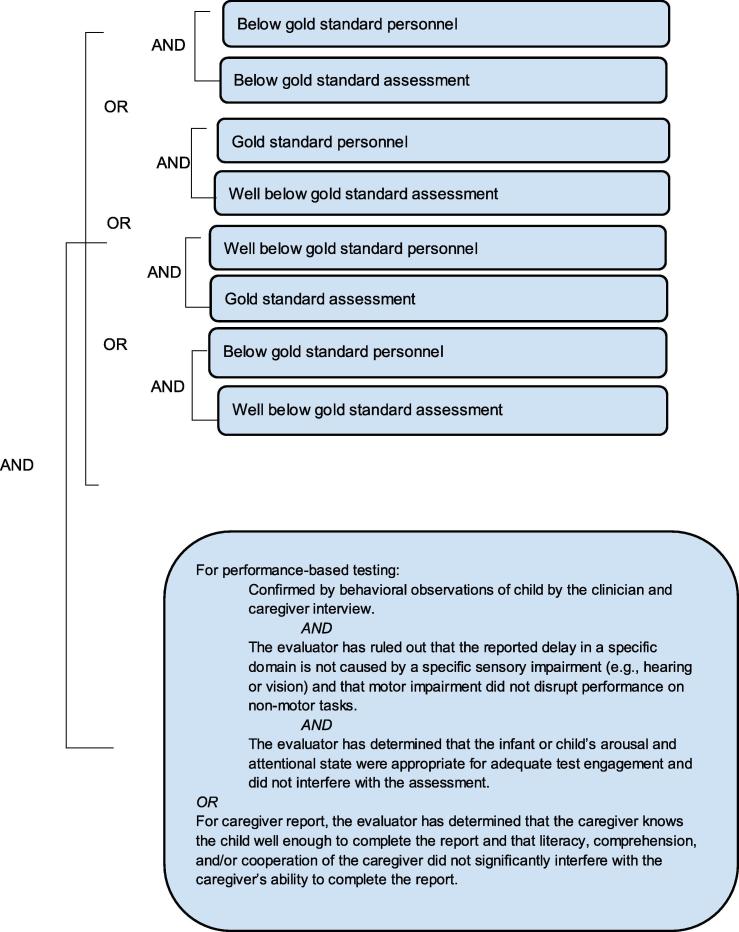
Diagnostic certainty.

**Level 3B:**
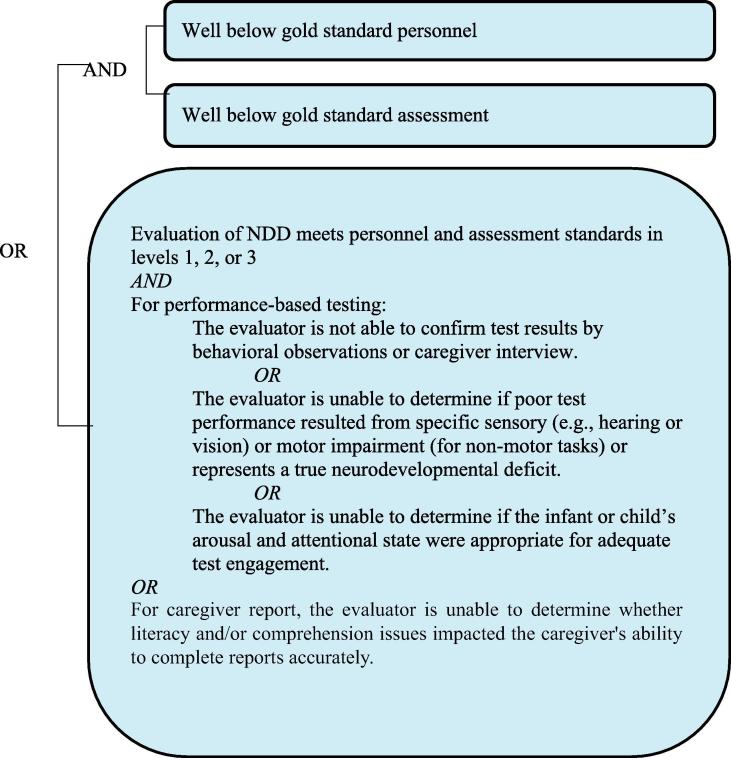
Diagnostic certainty.

The number of symptoms and/or signs documented for each case may vary considerably. The case definition has been formulated such that the Level 1 definition aims to be as specific as possible for the condition. As maximum specificity normally implies a loss of sensitivity, three diagnostic levels have been included in the definition, offering a stepwise attempt to increase sensitivity from Level 1 down to Level 3, while aiming to retain acceptable specificity at all levels.

#### 1.3.9 Consideration of premature birth

When the gestational age of a child is known, most tests require than an age correction is made to account for preterm birth. That is, the child is compared to children of the same chronological age minus the number of weeks born preterm. Guidelines for age corrections for prematurity are determined by the specific test being used and are generally made until 24  months of age. There is little empirical support for designating the appropriate length of time for which age corrections should be made and evaluators should abide by the guidelines set forth in the specific assessment being used [156].

Very often, the gestational age of a child is not known. In these cases, birth weight (if known or can be estimated) can often be used as a proxy for preterm birth utilizing a country-wide or regional data chart. However, in LMICs there are high rates of full-term, small for gestational age (SGA) babies born which may overestimate rates of preterm birth if these charts are used in isolation [157]. In these cases, the best available estimate should be used. The GAIA-Brighton Collaboration case definitions and LOC for preterm birth and SGA could be used as reference [158], [159].

#### 1.3.10 Consideration of sensory and motor impairments

Sensory (hearing or vision) and motor impairment do not preclude a diagnosis of NDD. In fact, they commonly co-occur with NDD given that the etiology of a sensory and/or motor impairment can also be the etiology of NDD (e.g., perinatal insult that results in visual impairment and cognitive delays). Additionally, significant sensory and motor impairments can also serve to limit a child’s ability to interact with the world around them, explore, and learn, further increasing the risk of development of NDD.

Among infants that survive prenatal, perinatal, or neonatal insult, approximately 18% have visual impairment, 20% have hearing impairment, and 17% have motor impairment [160]. Existing research highlights the complex interplay between visual impairment and early development [161], [162], [163]. Although there is individual variation in outcomes, studies identify between 21% and 71% comorbidity between visual impairment and developmental, learning, and medical problems [164]. Hearing impairment is also often related to cognitive functioning, with many etiologies of hearing loss related to risk for lower intelligence [165]. With regard to physical impairments, early motor delays often indicate broader neurological dysfunction [166], and there is a high rate of comorbidity between motor deficits and other NDD [167].

Despite the increased risk of NDD in these populations, children with known sensory or motor impairments are often excluded from the standardization samples of established developmental measures [168], and routine assessment of children with these impairments is not conducted in many countries [169]. Moreover, administration procedures for standardized developmental assessments are designed and established for children without these impairments. As a result there are often limited accommodations for children when these impairments are present [170].

Consideration of a sensory or motor impairment is always an essential part of a comprehensive neurodevelopmental assessment because NDD may be a clinical manifestation of a pathology also manifesting with sensory or motor impairments. Inquiring as to caregiver concerns for hearing, vision, or motor impairments and asking specific questions about sensory and motor behaviors is a critical aspect of evaluating for such problems early in development. Brief, behavioral screenings appropriate for the child’s age are conducted to assess the potential impact that limitations in any of these areas may have on the assessment. For example, hearing can be screened by observing behavioral responses to items on neurodevelopmental measures, such as response to novel sounds without visual input, response to commands or instructions, and response to questions. Vision can be screened by observing the child’s ability to fixate on, track, and localize objects, respond to items at a desktop distance, imitate visually presented behavior, complete puzzles, and match or sort objects. Motor skills screening, to ensure that a child has adequate motor skills to complete non-motor tasks, can include observing the child’s ability to hold, grasp, or reach for an object, maintain a sitting posture and head control, and point proximally. If concerns about a child’s hearing, vision, or motor skills arise, referral for a medical examination is warranted in addition to more in-depth hearing, vision, or motor examination. Additional information about medical examinations related to these concerns is beyond the scope of this paper.

Some test manuals provide possible accommodations for testing that do not alter test validity [115], [171]. Although for the majority of tests, research is lacking on the influence of adaptations of test administration on results [172]. Even when test manuals do not provide this guidance, an evaluator with expertise in 0–5 assessment may be able to make adaptations based on clinical judgment and experience and interpret the results meaningfully. For example, some subtests can be administered in standard format (e.g., a child with hearing impairment may be able to participate in motor assessment) and others can be adapted to minimize the impact of the sensory impairment (e.g., stimuli can be placed in the hand of a child with motor impairment to elicit exploration versus expecting the child to reach and grab). It may be determined that some tests cannot be administered validly due to the specific impairment (e.g., a child with visual impairment may not be able to engage in gross motor tasks, such as running or climbing stairs). An experienced evaluator is able to interpret test results with consideration of the sensory or motor impairment and the impact of any deviation from standardized administration.

### 1.4 Guidelines for data collection, analysis and presentation

The case definition is accompanied by guidelines that are structured according to the steps of conducting a clinical trial, i.e., data collection, analysis and presentation. Neither case definition nor guidelines are intended to guide or establish criteria for management of ill infants, children, or adults. Both were developed to improve data collection and comparability.

### 1.5 Periodic review

Similar to all Brighton Collaboration case definitions and guidelines, review of the NDD case definition with its guidelines is planned on a regular basis (i.e. every three to five years) or more often if needed.

## 2 Case definition of neurodevelopmental delay

### 2.1 For all levels of diagnostic certainty

Neurodevelopmental delay (NDD) is a term used to describe developmental functioning that is well below age expectations due to known or presumed central nervous system (CNS) dysfunction. NDD is identified during the early childhood developmental period which encompasses infancy, toddlerhood, and early childhood up to approximately 5  years of age. The current case definition operationally defines NDD as functioning at least two standard deviations below the norm in one or more of the following domains: gross motor, fine motor, expressive language, receptive language, and/or cognitive/problem-solving. The fundamental impact of a NDD is impairment in the child’s developmental course compared to peers of the same age [173].

While there is individual variability in “typical” neurodevelopment, the “normal distribution” is generally defined as the entire distribution of a set of characteristics that occur in a given population. For normally distributed outcomes, 68% of the population falls within one standard deviation of the mean and is considered to be functioning in the “average” range [8]. Again, for a normal distribution, 96% of the population falls within two standard deviations of the mean, with just 2% falling in each extreme tail of the distribution (i.e., well above or well below average). When results of developmental testing on standardized measures reach two standard deviations or more below the mean for age, these findings are considered significant and when deemed reliable within the context of gold standard levels of diagnostic certainty (LOC), correlated with higher risk and higher likelihood of NDD.

It should be noted, a delay that is 1.5 to < 2.0 standard deviations below the mean constitutes a high-risk area of concern for NDD and merits monitoring at a minimum. In some clinical settings in HICs, delays in this range are often classified and treated as NDD [174], [175]. In the context of research, documentation and on-going monitoring of this at-risk population through the use of the LOCs is strongly recommended.

Developmental surveillance through formal assessment is essential when a NDD has been detected. Given the variable nature of development and the broad variance in familial and cultural tolerance for developmental differences, assessment by a trained professional provides maximum certainty that a delay has not been present or does not arise in the period of birth to 5  years of age. Because some delays only become evident as children face increasing developmental demands, repeat assessment is necessary. As a result, we recommend that at least one assessment in each domain occur in at least one, or when feasible, in each of the following four time periods: early infancy (0–6 months), late infancy (7–18 months), toddlerhood (18–36 months) and preschool years (37–60 months).

The levels of diagnostic certainty below describe a single assessment time point for each individual domain. Therefore, for example, one could determine that a gross motor delay was or was not present with varying levels of certainty and that an expressive language delay was or not present with a different level of certainty during the first six months of life. The highest LOC in the diagnosis of NDD for each domain is achieved when gold standard personnel administer gold standard measures and assessment tools (see Table 3, Table 4, Table 5), wherein domains assessed within the gold standard LOC are more likely to yield certainty of true positives and minimize false negative findings of NDD. A decision tree is also provided to aid in the identification of the appropriate level of diagnostic certainty (see Appendix A).

**Table 5:** Levels of diagnostic certainty for NDD.

Personnel	Assessment
*Level 1*	
Gold standard	Gold standard
*Level 2*	
Gold standard	Below gold standard
Below gold standard	Gold standard
*Level 3A*	
Below gold standard	Below gold standard
Gold standard	Well below gold standard
Well below gold standard	Gold standard
Well below gold standard	Below gold standard
Below gold standard	Well below gold standard
*Level 3B*	
Well below gold standard	Well below gold standard
*Level 4*	
Reported NDD with insufficient evidence to meet the case definition	
*Level 5*	
Not a case of NDD	

## 3 Guidelines for data collection, analysis and presentation of neurodevelopmental delay

It was the consensus of the Brighton Collaboration *Neurodevelopmental Delay Working Group* to recommend the following guidelines to enable meaningful and standardized collection, analysis, and presentation of information about NDD. However, implementation of all guidelines might not be possible in all settings. The availability of information may vary depending upon resources, geographical region, and whether the source of information is a prospective clinical trial, a post-marketing surveillance or epidemiological study, or an individual report of a case of NDD. Also, these guidelines were developed by this working group for guidance only, and are not to be considered a mandatory requirement for data collection, analysis, or presentation.

### 3.1 Data collection

These guidelines represent a desirable standard for the collection of data on NDD in infants and young children following maternal immunization to allow for comparability of data, and are recommended as an addition to data collected for the specific study question and setting. The guidelines are not specifically intended to guide the primary reporting of neurodevelopmental delay to a surveillance system or study monitor, but they could potentially be adapted for these purposes. Investigators developing a data collection tool based on these data collection guidelines also need to refer to the criteria in the case definition, which are not repeated in these guidelines.

Guidelines numbered 1–42 below have been developed to address data elements for the collection of adverse event information as specified in general drug safety guidelines by the International Conference on Harmonization of Technical Requirements for Registration of Pharmaceuticals for Human Use, and the form for reporting of drug adverse events by the Council for International Organizations of Medical Sciences. These data elements include an identifiable reporter and patient, one or more prior maternal immunizations, and a detailed description of the adverse event, in this case, of NDD in infants following maternal immunization. The additional guidelines have been developed as guidance for the collection of additional information to allow for a more comprehensive understanding of neurodevelopmental delay in infants following maternal immunization.

#### 3.1.1 Source of information/reporter

For all cases and/or all study participants (including mothers and infants, as appropriate), the following information should be recorded:

(1) Date of report.
(2) Name and contact information of person reporting^2^ and/or diagnosing the neurodevelopmental delay as specified by country-specific data protection law.
(3) Name and contact information of the investigator responsible for the participant, as applicable.
(4) Relation to the patient (e.g., clinician, nurse, family member [indicate relationship], other).

#### 3.1.2 Vaccine/Control

##### 3.1.2.1 Demographics

For all cases and/or all study participants (including mothers and infants, as appropriate), the following information should be recorded:

(5) Case/study participant identifiers (e.g. first name initial followed by last name initial) or code (or in accordance with country-specific data protection laws).
(6) Date of birth, age (and corrected age to account for prematurity if used), and sex.
(7) For infants: Gestational age and birth weight

##### 3.1.2.2 Clinical and immunization history

For all cases and/or all study participants (including mothers and infants, as appropriate), the following information should be recorded:

(8) Past and current medical and obstetric history, including hospitalizations, underlying medical or neuropsychiatric diseases/disorders including cases of NDD in parents, siblings and/or close family members, maternal and infant nutritional status, pre-immunization signs and symptoms including identification of indicators for, or the absence of, a history of allergy to vaccines, vaccine components or medications; food allergy; allergic rhinitis; eczema; asthma. Other relevant information for this outcome may include maternal educational level, socioeconomic and environmental conditions.
(9) Any medication history (other than treatment for the event described) prior to, during, and after maternal immunization, including prescription and non-prescription medication as well as medication or treatment with long half-life or long-term effect. (e.g. immunoglobulins, blood transfusion and immunosuppressants), and alcohol or substance abuse.
(10) Maternal and infant immunization history (i.e. previous immunizations and any adverse event following immunization (AEFI))

#### 3.1.3 Details of the maternal and infant immunization

For all cases and/or all study participants (including mothers and infants, as appropriate), the following information should be recorded:

(11) Date and time of maternal and infant immunization(s).
(12) Description of vaccine(s) (name of vaccine, manufacturer, lot number, dose (e.g. 0.25 mL, 0.5 mL, etc.) and number of dose if part of a series of immunizations against the same disease).
(13) The anatomical sites (including left or right side) of all immunizations (e.g. vaccine A in proximal left lateral thigh, vaccine B in left deltoid).
(14) Route and method of administration (e.g. intramuscular, intradermal, subcutaneous, and needle-free (including type and size), other injection devices).
(15) Needle length and gauge.

#### 3.1.4 The adverse event

(16) For all cases at any level of diagnostic certainty and for reported events with insufficient evidence, the criteria fulfilled to meet the case definition should be recorded.

Specifically, document:

(17) Clinical description of signs and symptoms of NDD, and if there was medical confirmation of the event (i.e. patient seen by appropriate individual with expertise to confirm the diagnosis).
(18) Date/time of onset^3^, first observation^4^ and diagnosis^5^, duration and frequency of findings of NDD, last documented finding^6^ and final outcome^7^.
(19) Concurrent signs, symptoms, and diseases.
  • Measurement/testing – Values and units of routinely measured parameters (based on NDD testing tools) – in particular those indicating the severity of the event;
  • Method of measurement (e.g. type of assessor, type of measurement tool, date and duration of measurement, etc.);
  • Results of laboratory examinations (glucose, electrolytes, ultrasound…) surgical and/or pathological findings and diagnoses if present and pertinent.
(20) Treatment and/or interventions previously or currently implemented for NDD, especially specify if drug(s) are used and dosing.
(21) Outcome footnote 6 at last observation.
(22) Objective clinical evidence supporting classification of the event as “serious”^8^.
(23) Maternal and infant exposures other than the maternal immunization, including those 24 h before and after immunization, until delivery, and before and after the identification of the event (e.g. food, medications, environmental, etc.) considered potentially relevant to the reported event.

#### 3.1.5 Miscellaneous/general

• The duration of surveillance for NDD should be predefined based on the specific needs of the study.

Biologic characteristics of the vaccine (e.g. live attenuated versus inactivated component vaccines), biologic characteristics of the vaccine-targeted disease, biologic characteristics of the vaccine (e.g. nutrition, underlying disease like immune-depressing illness) may be relevant for the choice of the duration of the surveillance for Neurodevelopmental Delay.

(24) The duration of follow-up reported during the surveillance period should be predefined. It should aim to continue to resolution of the event, or its stabilization, as pertinent.
(25) Methods of data collection should be consistent within and between study groups, if applicable.
(26) Follow-up of cases should attempt to verify and complete the information collected as outlined in data collection guidelines 1–24.
(27) Investigators of patients with NDD should provide guidance to reporters to optimize the quality and completeness of information provided.
(28) Reports of NDD should be collected throughout the study period regardless of the time elapsed between maternal or infant immunization and the adverse event. If this is not feasible due to the study design, the study periods during which safety data are being collected should be clearly defined.

### 3.2 Data analysis

The following guidelines represent a desirable standard for analysis of data on NDD to allow for comparability of data, and are recommended as an addition to data analyzed for the specific study question and setting.

(29) Reported events should be classified in one of the following five categories including the three LOC. Events that meet the case definition should be classified according to the LOC as specified in the case definition. Events that do not meet the case definition should be classified in the additional categories for analysis.

**Event classification in 5 categories**^9^

**Event meets case definition**

(1) Level 1: Criteria as specified in the NDD case definition
(2) Level 2: Criteria as specified in the NDD case definition
(3) Level 3: Criteria as specified in the NDD case definition

**Event does not meet case definition**

***Additional categories for analysis***

(4) Level 4: Reported NDD with insufficient evidence to meet the case definition^10^
(5) Level 5: Not a case of NDD^11^
(30) The interval between maternal immunization and reported NDD could be defined as the date/time of maternal immunization to the date/time of onset footnote 2 of the first symptoms and/or signs consistent with the definition. Additionally, the occurrence of NDD in relation to the infant’s age should be reported. If few cases are reported, the concrete time course could be analyzed for each; for a large number of cases, data can be analyzed in the following increments based on trimester of maternal immunization, or infant’s age:

Subjects with Neurodevelopmental Delay by interval to presentation in relation to trimester of maternal immunization and age of the child.

**Table:** 

Interval*	Number
*In relation to maternal vaccination*	
First trimester	
Second trimester	
Third trimester	
Any time during pregnancy	
*In relation to infant or child age*	
0–6 months of age	
7–12 months	
13–36 months of age	
37–60 months of age	
After 5 years of age	
TOTAL

(31) The duration of NDD could be analyzed as the interval between the date/time of onset footnote 1 of the first symptoms and/or signs consistent with the definition and the last evaluation footnote 5 and/or final outcome footnote 6. Persistence beyond the last evaluation should be noted. Whatever start and ending times are used, they should be used consistently within and across study groups.
(32) If more than one measurement of a particular criterion is taken and recorded, the value corresponding to the greatest magnitude of the adverse experience could be used as the basis for analysis. Analysis may also include other characteristics like qualitative patterns of criteria defining the event.
(33) The distribution of data (as numerator and denominator data) could be analyzed in predefined increments (e.g. measured values, times), where applicable. Increments specified above should be used. When only a small number of cases are presented, the respective values or time course can be presented individually.
(34) Data on NDD obtained from subjects born to mothers receiving a vaccine should be compared with those obtained from an appropriately selected and documented control group(s) to assess background rates of hypersensitivity in non-exposed populations, and should be analyzed by study arm and dose where possible, e.g. in prospective clinical trials.

### 3.3 Data presentation

These guidelines represent a desirable standard for the presentation and publication of data on NDD in infants following maternal immunization to allow for comparability of data, and are recommended as an addition to data presented for the specific study question and setting. Additionally, it is recommended to refer to existing general guidelines for the presentation and publication of randomized controlled trials, systematic reviews, and meta-analyses of observational studies in epidemiology (e.g. statements of Consolidated Standards of Reporting Trials (CONSORT), of Improving the quality of reports of meta-analyses of randomized controlled trials (QUORUM), and of Meta-analysis Of Observational Studies in Epidemiology (MOOSE), respectively) [176].

(35) All reported events of NDD should be presented according to the categories listed in guideline 30 or other classification that is considered appropriate.
(36) Data on possible NDD events should be presented in accordance with data collection guidelines 1–28 and data analysis guidelines 29–34.
(37) Terms to describe NDD such as “low-grade”, “mild”, “moderate”, “high”, “severe” or “significant” are highly subjective, prone to wide interpretation, and should be avoided, unless clearly defined.
(38) Data should be presented with numerator and denominator (n/N) (and not only in percentages), if available.

Although NDD safety surveillance systems denominator data are usually not readily available, attempts should be made to identify approximate denominators. The source of the denominator data should be reported and calculations of estimates be described (e.g. manufacturer data like total doses distributed, reporting through Ministry of Health, coverage/population based data, etc.).

(39) The incidence of cases in the study population should be presented and clearly identified as such in the text.
(40) If the distribution of data is skewed, median and range are usually the more appropriate statistical descriptors than a mean. However, the mean and standard deviation should also be provided.
(41) Any publication of data on NDD in infants after maternal immunization should include a detailed description of the methods used for data collection and analysis as possible. It is essential to specify:
  • The study design;
  • The method, frequency and duration of monitoring for NDD;
  • The trial profile, indicating participant flow during a study including drop-outs and withdrawals to indicate the size and nature of the respective groups under investigation;
  • The type of surveillance (e.g. passive or active surveillance);
  • The characteristics of the surveillance system (e.g. population served, mode of report solicitation);
  • The search strategy in surveillance databases;
  • Comparison group(s), if used for analysis;
  • The instrument of data collection (e.g. standardized questionnaire, diary card, report form);
  • Whether the date of onset footnote 2 and/or the date of first observation footnote 3 and/or the date of diagnosis footnote 4 was used for analysis; and
  • Use of this case definition for NDD, in the abstract or methods section of a publication^12^.

## 4 Disclaimer

The findings, opinions and assertions contained in this consensus document are those of the individual scientific professional members of the working group. They do not necessarily represent the official positions of each participant’s organization (e.g., government, university, or corporation). Specifically, the findings and conclusions in this paper are those of the authors and do not necessarily represent the views of their respective institutions.

## Declaration of Competing Interest

The authors declared that there is no conflict of interest.

## Footnotes

9 The highest level of diagnostic certainty achieved for each domain should be recorded.

## Footnotes

2 If the reporting center is different from the vaccinating center, appropriate and timely communication of the adverse event should occur.

3 The date and/or time of onset is defined as the time when the first sign or symptom indicative for NDD occurred. This may only be possible to determine in retrospect.

4 The date and/or time of first observation of the first sign or symptom indicative for NDD can be used if date/time of onset is not known.

5 The date of diagnosis of an episode is the day when the event met the case definition at any level.

6 The end of the occurrence of NDD, related to spontaneous recovery in early childhood and/or positive response to treatment/intervention, is defined as the time the subject no longer meets the case definition at the lowest level of the definition.

7 E.g. recovery to pre-event health status, spontaneous resolution, therapeutic intervention, persistence of the event, sequelae, death.

8 An AEFI is defined as serious by international standards if it meets one or more of the following criteria: 1) it results in death, 2) is life-threatening, 3) it requires inpatient hospitalization or results in prolongation of existing hospitalization, 4) results in persistent or significant disability/incapacity, 5) is a congenital anomaly/birth defect, 6) is a medically important event or reaction.

10 If the evidence available for an event is insufficient because information is missing, such an event should be categorized as “Reported Neurodevelopmental Delay with insufficient evidence to meet the case definition”.

11 An event does not meet the case definition if investigation reveals a negative finding of a necessary criterion (necessary condition) for diagnosis. Such an event should be rejected and classified as “Not a case of Neurodevelopmental Delay”.

12 Use of this document should preferably be referenced by referring to the respective link on the Brighton Collaboration website (http://www.brightoncollaboration.org).
