# Supplementary material for: Neurodevelopmental delay: Case definition & guidelines for data collection, analysis, and presentation of immunization safety data
Source: Vaccine. 2019 Dec 10;37(52):7623–41. doi: 10.1016/j.vaccine.2019.05.027 (PMC6899448; doi:10.1016/j.vaccine.2019.05.027)
Supplement: Supplementary data 1 [file mmc1.docx]

# APPENDIX A: Tool to aid identification of appropriate level of diagnostic certainty for Neurodevelopmental Delay

1. Is there personnel and a test available to evaluate the child?
   1. If yes, skip to question 4.
   2. If no, proceed to question 2.
2. Is there personnel and a universal or regional developmental milestones checklist available?
   1. If yes, skip to question 3.
   2. If no, STOP. NDD is not diagnosed.
3. Is there a delay when measured against a universal or regional developmental milestone checklist that is confirmed by behavioral observation and reliable caregiver report?
   1. If no 🡪 STOP. NDD is not diagnosed.
   2. If yes, 🡪 Apply a LOC 3A or 3B depending on personnel qualifications.
4. Was the evaluator able to confirm test results by behavioral observation and reliable caregiver report?
   1. If no🡪 Apply a LOC 3B.
   2. If yes, proceed to question 5.
5. Was the evaluator able to rule out that the observed delay in a specific domain was not caused by a sensory impairment (e.g. hearing or vision) and that motor impairment did not disrupt performance on non-motor tasks?
   1. If no🡪 Apply a LOC 3B.
   2. If yes, proceed to question 6 for performance-based testing. Proceed to question 7 for caregiver report.
6. For performance-based testing, was the evaluator able to determine that the child’s arousal and attentional state were appropriate for adequate test engagement?
   1. If no🡪 Apply a LOC 3B.
   2. If yes, proceed to question 8.
7. For caregiver report, was the evaluator able to determine that literacy and/or comprehension issues did not interfere with the caregiver’s ability to complete reports accurately?
   1. If no🡪 Apply a LOC 4.

If yes, apply a LOC 3A or 3B depending on personnel level.

1. Select the appropriate level for personnel and tests and then classify according to level.

**Levels of diagnostic certainty:**

| **Level** | **Personnel** | **Test** |
| --- | --- | --- |
| 1 | Gold standard | Gold standard |
| 2 | Gold standard | Below gold standard |
| 2 | Below gold standard | Gold standard |
| 3A | Below gold standard | Below gold standard |
| 3A | Gold standard | Well below gold standard |
| 3A | Well below gold standard | Gold standard |
| 3A | Well below gold standard | Below gold standard |
| 3A | Below gold standard | Well below gold standard |
| 3B | Well below gold standard | Well below gold standard |
| 4 | Reported NDD with insufficient evidence to meet the case definition | |
| 5 | Not a case of NDD | |
